# Effect of Subanaesthetic Dose of Ketamine on Pneumoperitoneal Response and Clinical Recovery in Patients Undergoing Laparoscopy

**DOI:** 10.5152/TJAR.2022.21066

**Published:** 2022-06-01

**Authors:** Swaminathan Veerasamy Rajarajan, Arun Kumar Alarasan, Anand Subramaniam, Lailu Mathews

**Affiliations:** 1 Department of Anaesthesiology, Chettinad Hospital and Research Institute, Kelambakkam, Chennai, India

**Keywords:** Hemodynamics, ketamine, laparoscopic, pain, postoperative, recovery

## Abstract

**Objective:**

Although suppression of intraperitoneal gas insufflation response is possible with a higher dose of opioids, sedatives, and inhalational agents, delayed emergence and poor clinical recovery are still a matter of concern. Here our primary aim was to assess the quality of recovery and the secondary aim includes postinsufflation response, postoperative pain intensity, total opioid requirement, and looking for adverse effects, if any.

**Methods:**

This prospective randomized double-blinded controlled study was conducted among 75 American Society of Anesthesiologist physical status I and II patients scheduled for laparoscopic surgeries under general anaesthesia. Group 1 received injection tramadol 1 mg kg^−1^ iv^−1^ 5 minutes after intubation. Similarly, groups 2 and 3 received 0.25 mg kg^−1^ and 0.5 mg kg^−1^ injection of ketamine iv, respectively. Intraperitoneal insufflation response was observed from the beginning of insufflation till 15 minutes. Clinical recovery was measured in terms of vigilance, cognition, orientation, and comfort. Postoperative pain intensity was assessed at varying movement activities using numerical rating scale pain score and with the total opioid requirement. The collected data were analyzed using three-way ANOVA.

**Results:**

Groups 1 and 2 had a fair clinical recovery. Postoperative pain intensity was least in group 2, and the postinsufflation mean arterial pressure was higher in groups 1 and 3. A total of 32% of participants had delirium in group 3.

**Conclusions:**

Clinical recovery and perioperative analgesia were better in ketamine group (0.25 mg kg^−1^) without any perturbations in intra-operative pneumoperitoneal response. Hence it can be considered an optimal adjuvant in laparoscopic surgeries.

**Main Points**

- Attenuation of pneumoperitoneal insufflations response with higher doses of opioids, sedatives, and inhalational agents results in delayed emergence and poor clinical recovery in laparoscopic surgeries.
- Preemptive analgesic effect of ketamine is well known to decrease the total opioid requirement and need for rescue analgesia.
- Its role in pneumoperitoneal response and postextubation clinical recovery is still inconclusive.
- Fair clinical recovery was found in group 2 without any untoward perturbations in pneumoperitoneal response.

## Introduction

During laparoscopy, the post-inflation increase in intraabdominal pressure of 15 mmHg was associated with an increase in systemic vascular resistance by 65%, mean arterial pressure by 35%, pulmonary vascular resistance by 90%, and a fall in cardiac index by 20%.^1^

Attenuation of this insufflation response with a high dose of opioids, sedatives, nitroglycerin,^2^ and inhalational agents was accompanied by delayed emergence and clinical recovery. Recent studies quote that time taken to eye opening from discontinuation of general anaesthesia was significantly delayed with co-administration of dexmedetomidine compared to that of clonidine.^3^

Perioperative noxious stimuli cause prolonged firing of c-fiber nociceptors resulting in activation of postsynaptic N-methyl D-aspartate receptor (NMDA). Stimulated NMDA receptors lead to pain processing, which in turn contributes to inflammation, neuropathic pain, and secondary hyperalgesia.^4^ Analgesic intervention before noxious stimuli may attenuate or block sensitization, thereby reducing acute pain.^5^

Tramadol and ketamine are well known for their preemptive analgesic effect in a variety of surgeries.^6,7^ Low-dose tramadol (0.5-1 mg kg^−1^ iv^−1^) was shown as an effective pre-emptive analgesic agent in pediatric tonsillectomy and laparoscopic surgeries.^8,9^ In one clinical trial, 0.5 mg kg^−1^ of IV ketamine was found to provide better preemptive analgesia than 0.5 mg kg^−1^ of rectal ketamine and 1 mg kg^−1^ of IV tramadol.^10^ Recent research on the analysis of cytokine changes with low-dose ketamine illustrated that 0.25 mg kg^−1^ ketamine was sufficient to decrease the secretion of inflammatory mediators, thereby attenuating postoperative pain and opioid requirement.^11^

Subanaesthetic dose of ketamine was found to blunt the hemodynamic response to skull pin placement in one study.^12^ But their influence on postinsufflation response and clinical recovery in laparoscopic surgeries is very much limited. Hence our primary outcome was to assess the quality of recovery following extubation, and the secondary outcome includes postinsufflation hemodynamic response, postoperative pain intensity, total opioid requirement, and look for side effects, if any.

## Methods

This prospective randomized double-blinded study was conducted in the department of anaesthesiology following approval from the institutional human ethics committee and registration at the clinical trial registry [CTRI no: CTRI/2019/04/018422], India. It was carried out over a period of 7 months.

American Society of Anesthesiologist physical status (ASA) I and II patients of age group 18-60 years with good cognitive function and without any sensorineural deafness were included in this study. Only laparoscopic cholecystectomy and appendicectomy were included. Participants with (Body Mass Index) BMI > 30 kg m^−2^, those with psychiatric illness, those who are allergic to study drugs, and pregnant females were excluded from this study. After obtaining well-informed written consent, the study population was randomly divided into 3 groups of 25 each. Group allocation was done with the help of prefilled coded syringes wrapped in an opaque sheet of paper. Randomization was carried out via computer-generated randomization code: group 1 (n = 25) received injection tramadol 1 mg kg_−1_ iv_−1_, group 2 (n = 25) received injection ketamine 0.25 mg kg_−1_ iv_−1_, group 3 (n = 25) received injection ketamine 0.5 mg kg_−1_ iv_−1_.

Tramadol was chosen as an active placebo in the control group in order to overcome ethical issues. After thorough preanaesthetic evaluation, all patients were allowed to fast for 6 hours prior to surgery and concomitant Ringer lactate intravenous infusion was started and maintained as per kilogram body weight to avoid overnight dehydration. All were premedicated with tablet diazepam 5 mg and tablet ranitidine 150 mg on the morning of surgery. After shifting the patient to the operation theater, hemodynamic variables like heart rate, blood pressure, respiratory rate, and oxygen saturation were monitored. Injection midazolam 1 mg and injection glycopyrolate 0.2 mg were given intravenously 5 minutes before induction.

All were preoxygenated with 100% O_2_ for 3 minutes and anaesthesia was induced with injection of propofol 2 mg kg^−1^ and fentanyl 2 µg kg^−1^ intravenously. After checking for adequate ventilation, neuromuscular blockade with injection of atracurium 0.5 mg kg^−1^ was given and the trachea was intubated with an appropriate size endotracheal tube. The study drug was loaded as per kilogram body weight and diluted to 10 mL in a 10 mL syringe labeled with the appropriate code. It was prepared by an anaesthesiologist not involved in the study. The study drug was injected intravenously slowly over a period of 5 minutes, 5 minutes after intubation prior to skin incision by a senior anaesthesiologist not involved in the study. The investigator and the person injecting the drug were both blinded about the study drug.

All were mechanically ventilated on volume-controlled ventilation mode and anaesthesia was maintained with oxygen and air in a ratio of 1:1 along with isoflurane upto 1.5% to achieve 1 Minimal Alveolar Concentration (MAC). Neuromuscular blockade was maintained with injection of atracurium 0.1 mg kg^−1^ iv^−1^. Injection of paracetamol 1 g and fentanyl 1 μg kg^−1^ h^−1^ were given intravenously to attain intra-operative analgesia in all patients. Based on the evidence of untoward consequences of high intraabdominal pressure in previous studies,^1^ pneumoperitoneum was created with CO_2_ at an insufflation pressure of 12-14 mm Hg in our study and postinsufflation monitoring of heart rate, mean arterial pressure, and airway pressure was done at baseline, 2-minute interval upto 10 minutes and then at 15 minutes. Postinsufflation response was explained by tachycardia, hypertension, and a rise in peak airway pressure. In case the surgery could not be done under laparoscopy, it was converted to an open procedure and those patients were excluded from the study.

At the end of the surgery, reversal of neuromuscular blockade was accomplished with intravenous administration of injection Neostigmine 0.04 mg kg^−1^ and glycopyrrolate 0.01 mg kg^−1^, and extubation was done once adequate efforts of spontaneous respiration and consciousness were met.

Post extubation, all patients were transferred to post-anaesthesia recovery room, where the assessment of recovery from anaesthesia was done by an anaesthesiologist not involved in the study, using a clinical recovery score.^13^ The clinical recovery score was assessed using 4 parameters: (a) Vigilance—0-unconscious, not arousable, 1-unconscious, arousable by noxious stimuli, 2-unconscious, arousable by verbal stimuli, 3-drowsy, 4-awake, not attentive, 5-awake, attentive; (b) Cognition—0-no understanding of simple words, 1-good understanding of simple words; (c) Orientation—0-confused, 1-disoriented, 2-well oriented; (d) patient’s condition—1-uncomfortable, 2-comfortable, 3-excellent. Scores: 11-excellent recovery, 9-10-good recovery, 8-fair recovery, <8-poor recovery.

Postoperatively, all enrolled patients were observed for pain using a numerical rating scale for pain^14^ from 0, 2, 4, 6, 8, 10, 12, and 24 hours. The time 0 hour was taken as the baseline value upon entering the recovery room. On each observation, pain intensity was evaluated at rest, at slight movement of breathing, and at deep breathing by a trained nurse (who was blinded about the study drug). These observations were made in the recovery room and in the ward. All patients received injection of paracetamol 1 gram iv 8 hourly as the basal analgesic and when pain score is >4, Morphine 2 mg was used as the rescue analgesic. After 24 hours, analgesics were given at the discretion of anaesthesiologist/surgeon.

Time of the first request of supplemental analgesia, the total number of patients demanding supplemental analgesia, and the total opioid dose consumed in the first 24 hours in the postoperative period were documented. Postoperative monitoring of vital parameters was done at 0, 2, 4, 6, 12, and 24 hours respectively in the post-anaesthesia care unit. Adverse effects, if any, associated with ketamine, such as nausea, vomiting, and delirium, were also recorded.

Tachycardia was defined as an increase in heart rate >100 beats per minute, and hypertension was taken as the rise in mean arterial pressure 20% above the baseline value. Rise in peak airway pressure was explained by an increment in peak airway pressure >40% above the baseline airway pressure at an insufflation pressure of 14 mm Hg of CO_2_.

### Statistical Analysis

The sample size was calculated using G*Power statistical software using a scenario to detect the difference between k means by one-way analysis of variance (ANOVA) with fixed effects using F statistic. Assuming a least mean recovery score of 7.5 with a minimal clinically important difference as 0.5 units with an assumed standard deviation of 1 and 3 study groups with equal sample size, the effect size was calculated as 0.408 from the pilot study. With this effect size, assuming 80% power and 5% two-sided alpha error, the required sample size was 63 with 21 subjects in each group. To account for non-participation rate and loss of follow-up, we have included 25 subjects in each group.

The collected data were analyzed using three-way ANOVA in Statistical Package for Social Sciences ver. 21 (IBM Corp.; Armonk, NY, USA) and intergroup comparison was done by student t-test. *P* < .05 was considered statistically significant.

## Results

**Figure 1.**
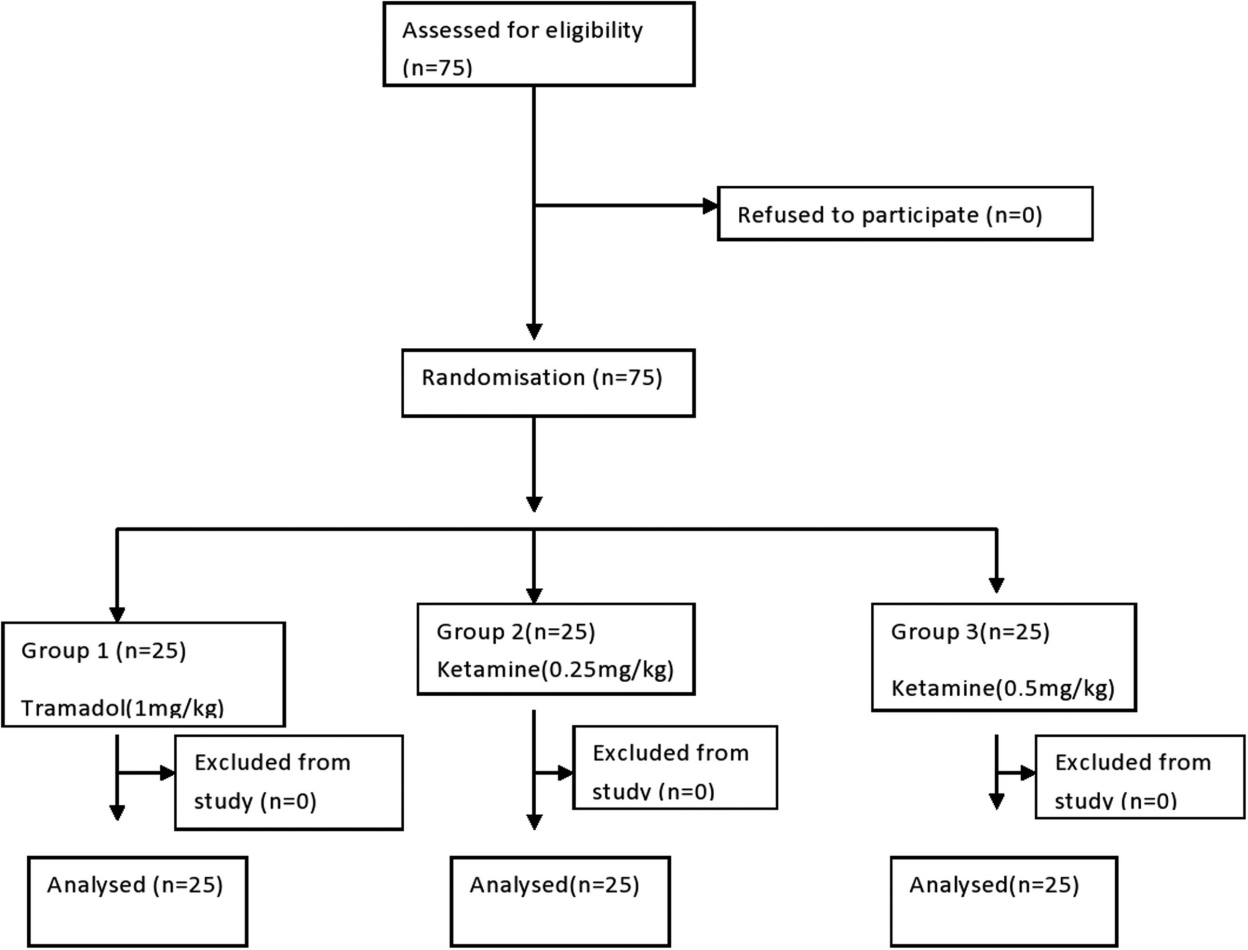
Randomization flow chart.

All the 75 patients who were recruited in this study were willing to participate, and they cooperated till the end of this study. There were no defaulters (Figure 1). There was no statistically significant difference in the demographic characteristics and duration of surgery of the study population (Table 1).

Intraoperatively, postinsufflation heart rate response was stable in all the 3 groups throughout the study period. In contrast, the trend in postinsufflation mean arterial pressure was higher in group 1 and group 3 throughout the study period compared to group 2. But their intergroup comparison was not statistically significant. Immediately after gas insufflation, there was a slight increase in peak airway pressure in all the groups which was nearly 20% above the baseline value. But none of them reached >40%.

The mean clinical recovery score observed at 0 hour was (8.8 ± 1.2 ) in group 1 and (8.7 ± 0.3) in group 2, and it was significantly low in group 3 (6.2 ± 0.2) (*P* = .019).

**Figure 2.**
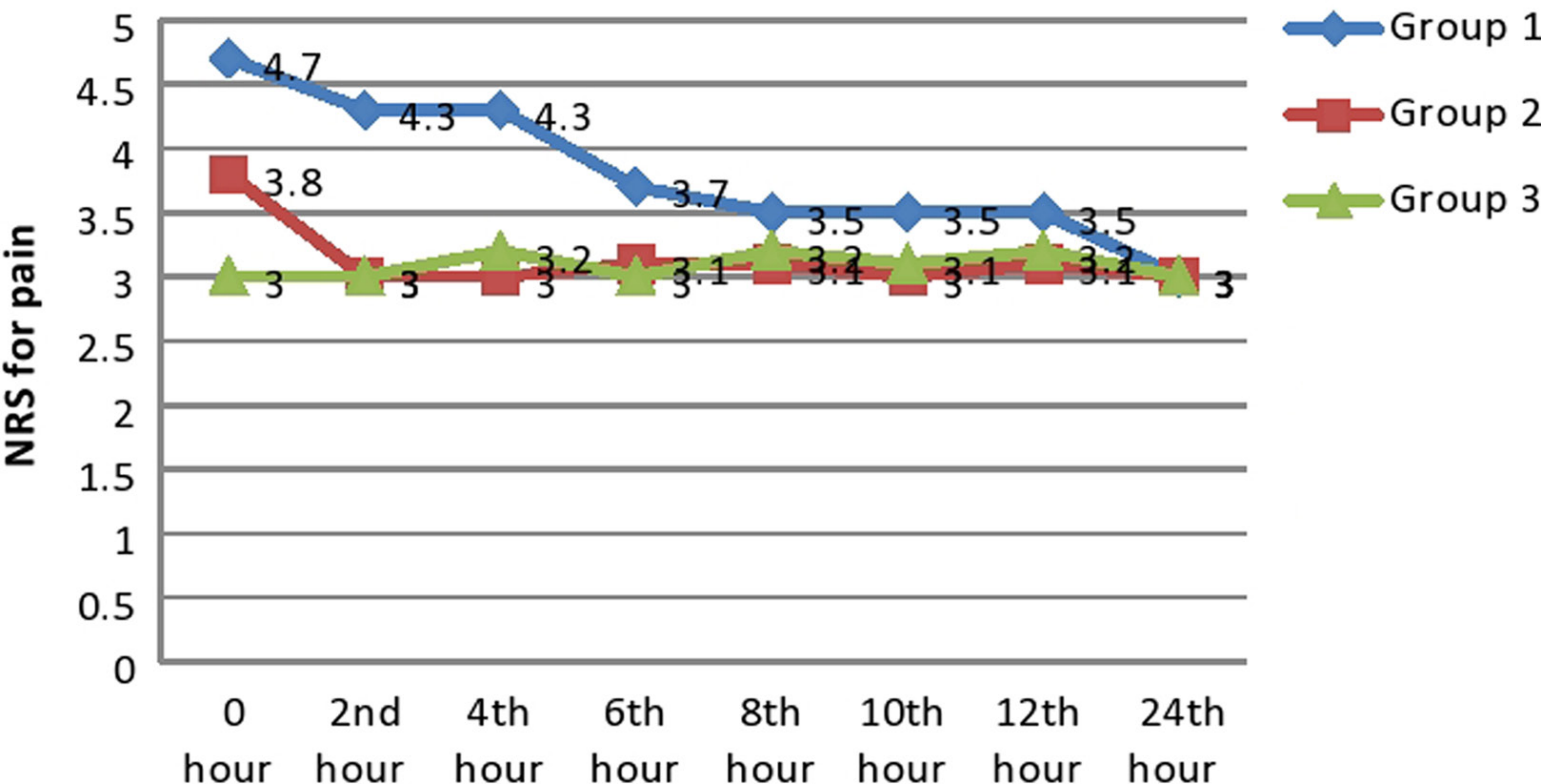
Distribution of pain score at rest among the study groups.

**Figure 3.**
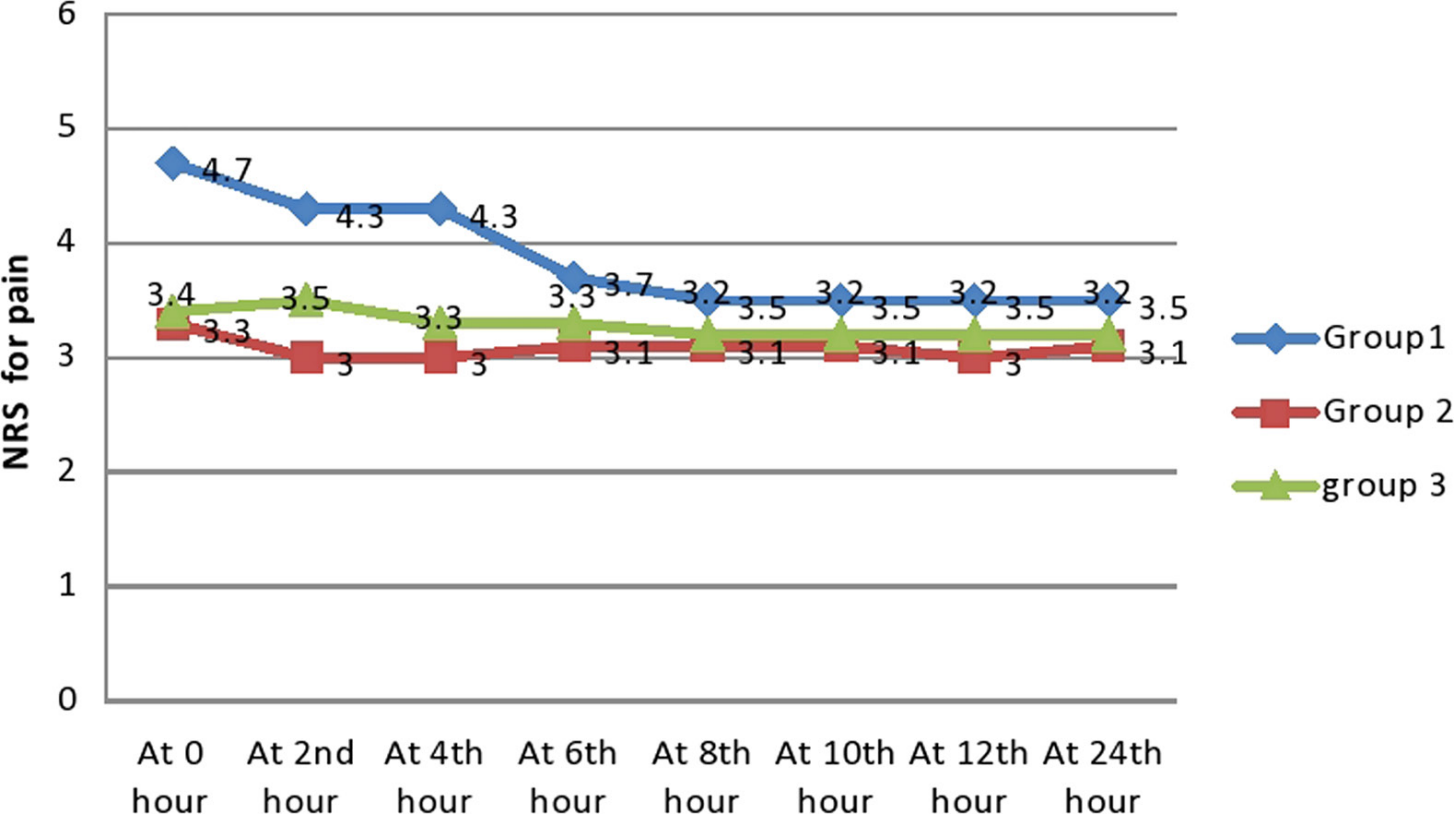
Distribution of pain score at slight movement among study groups.

Postoperatively the mean numerical rating scale (NRS) for pain score at rest, at slight movement of breathing, and at deep breathing was less than 5 in all the 3 groups. At 0 hour, the pain score at rest (Figure 2) and at slight breathing movement was higher in group 1 (pain score > 4). Groups 2 and 3 had a lesser pain score at rest and at slight breathing movement (<4) compared to group 1 throughout the study period. Until 2 hours, there was a statistically significant reduction in pain score between groups 2 and 3 (*P* = 0.008) (Figure 3).

**Figure 4.**
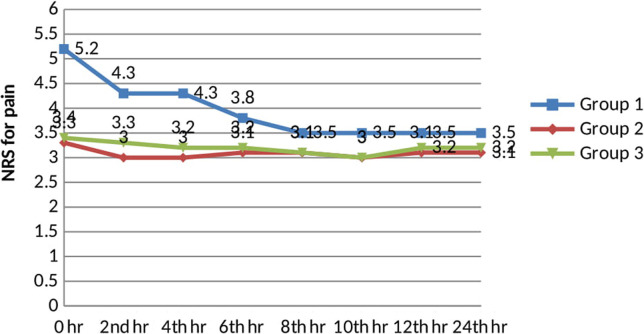
Distribution of pain score at deep breathing among study groups.

Similarly, pain score for deep breathing movement was high in group 1 (pain score > 4) even at 0 hour (Figure 4). Groups 2 and 3 had lesser pain score (<4) compared to group 1 throughout the study period. It was statistically insignificant. Postoperative morphine requirement was higher in group 1 (3.6 ± 0.5) compared to group 2 (0.6 ± 0.3) and group 3 (1.5 ± 0.0). At 60-120 minutes, there was a significant difference in the requirement of morphine in group 2 and group 3 (*P* = .037).

Incidences of nausea and vomiting were high in group 1 (20% and 12%) and group 3 (20% and 8%) compared to group 2 (8% and 8%), respectively. But delirium seemed to be higher in group 3 (32%) compared to group 2 (12%) (Table 2). There was no incidence of delirium in group 1.

## Discussion

Our study aimed to test the hypothesis that 0.25 mg kg^−1^ iv^−1^ ketamine was superior to 0.5 mg kg^−1^ iv^−1^ ketamine and 1 mg kg^−1^ iv^−1^ tramadol in terms of postinsufflation hemodynamic response and better clinical recovery in patients undergoing laparoscopic abdominal surgeries.

Results of the study concluded that groups 1 and 2 had a fair clinical recovery. Postinsufflation response and postoperative pain intensity were least in group 2. Total opioid requirement for rescue analgesia was more in group 1 and side effects like delirium, nausea, and vomiting were common in group 3.

Subanaesthetic doses of ketamine (0.2 mg kg^−1^ or 0.4 mg kg^−1^) did not improve the quality of recovery after remifentanyl-based anaesthesia for laparoscopic surgeries in a study done by Moro et al.^15^ Recovery of cerebral function following ketamine anaesthesia with racemic ketamine mixture (1.3 mg kg^−1^) and (S) ketamine (0.65 mg kg^−1^) was compared among 12 healthy volunteers in a cross-over study using neuropsychological test. In addition, physostigmine was added to S-ketamine group to test its antagonizing efficacy. They concluded that S-ketamine had a shorter recovery time compared to the racemic mixture and the mean time to reach preoperative test performance was 117.5 minutes in S-ketamine/physostigmine, 121.3 minutes in S-ketamine/NaCl, and 141.6 minutes in racemic ketamine.^16^ Despite being a racemic mixture, fair clinical recovery was observed in groups 1 and 2 at 0 hour in our study.

In one literature, ketamine–propofol combination used at a concentration of 8 mg mL^−1^ for puerpural sterilization was found to provide a better recovery profile than propofol–fentanyl combination in terms of maternal comfort in handling the baby and ability to breastfeed the baby.^17^ White et al^18^ evaluated the brain and plasma concentrations of ketamine in rats following either intramuscular injection alone or in combination with halothane anaesthesia. They noted that rats with halothane anaesthesia had double the ketamine concentration in brain than those with ketamine alone. They concluded that half-life of ketamine in plasma and brain was doubled with halothane anaesthesia. Similar studies with isoflurane haven’t been done. Although 40-item quality of recovery scoring system (QOR-40), Aldrete scores, modified Aldrete scores, and so on were available for assessment of recovery from anaesthesia, clinical recovery score based on 4 parameters was chosen in our study because of its simplicity.

Postinsufflation hemodynamic responses to laparoscopy were least in group 2.

Jee et al^19^ in their study on pneumoperitoneal response to magnesium sulfate infusion (MgSO_4_) during laparoscopy demonstrated that intravenous MgSO_4_ decreased the release of catecholamines and vasopressin in response to stress and also attenuated the arterial pressure response to laparoscopy. Similar reports with low-dose ketamine (0.25 mg kg^−1^) have been noticed with tourniquet induced arterial pressure response in orthopedic surgeries under general anaesthesia.^20^ Both being NMDA antagonists, our study results confirm to it.

NRS for pain at rest, at slight breathing movement, and at deep breathing were high (>4) even at 0 hour in group 1 owing to poor analgesia with a weak opioid. At the similar type of body movements, groups 2 and 3 had lesser pain scores throughout the study period. It could be attributed to the preemptive analgesic effect of low-dose ketamine. Till 2nd hour of postoperative observation, group 2 had better pain relief and their intergroup comparison with group 3 was significant. In support of this finding, Kartalov et al^11^ demonstrated that 0.25 mg kg^−1^ ketamine was sufficient to decrease the secretion of TNF-α, IL-1β, and IL-6, thereby attenuating postoperative pain and opioid requirement.

Moreover higher doses of ketamine (500 µg kg^−1^) were known to induce impairment of cognitive functions such as attention, free recall, recognition memory, and thought process, in previous studies.^21^ In accordance, the cognitive dysfunction effect was noted in group 3 while responding to NRS in our study and could account for the significant difference in intergroup comparison postoperatively. On subsequent analysis by Singh et al.^22^ 0.5 mg kg^−1^ iv^−1^ ketamine was shown to be equally efficacious as 0.75 mg kg^−1^ iv^−1^ and 1 mg kg^−1^ iv^−1^ ketamine in decreasing visual analog scale (VAS) and verbal rating scale (VRS) at rest, a slight movement and deep breathing movement.

Total morphine requirement for rescue analgesia was high in group 1 (3.6 ± 0.5) compared to group 2 (0.6 ± 0.3) and group 3 (1.5 ± 0.0). But this was in contradiction with the study done by Wordliczek et al^23^ where preemptive analgesic effect of tramadol significantly decreased postoperative opioid requirement.

At around 60-120 minutes, there was a significant difference in the requirement of morphine in group 2 and group 3. Administration of low dose of ketamine before skin incision has the property of attenuating acute tolerance and hyperalgesia induced by co-administered opioid.^24^ Smaller the dose, the greater the affinity of ketamine for NMDA receptors. Low-dose ketamine was defined by a serum concentration of 30-120 ng mL^−1^.^25^ Prior administration of small dose of ketamine (75-150 µg kg^−1^) was proven to reduce postoperative opioid consumption in previous literatures.^26^ This could be the reason for the lesser requirement of morphine as rescue analgesia in groups 2 and 3 in our study. In the contrary, Ithnin et al^27^ illustrated that a low dose of S-ketamine doesn’t decrease the total morphine requirement for rescue analgesia.

In our study, the incidence of nausea and vomiting was high in group 1 (20% and 12%) and group 3 (20% and 8%). Singh et al^22^ in their study observed that the occurrence of nausea and vomiting was equally present in all doses of ketamine ranging from 0.5 mg kg^−1^ to 1 mg kg^−1^. But Chandrakantan et al^28^ in their trial on postoperative nausea and vomiting (PONV) declared that 0.25 mg kg^−1^ ketamine was associated with decreased incidence of nausea and vomiting. Delirium was found to be higher in group 3 (32%). This wasn’t in accordance with the findings of previous literatures where delirium was found only at a dose of 1 mg kg^−1^ ketamine.^22^ The premedication dosage of tablet diazepam and injection midazolam was standardized and not weight-based in our study. The timing of administration of the study drug was 30 minutes prior to the incision in previous study by Singh et al^22^ and 5 minutes prior to skin incision in our study. Both these factors might have contributed to a higher incidence of delirium in group 3.

Our study also had some limitations. We did not follow up with the patient for any development of chronic pain and the serum level of 0.25 mg kg^−1^ ketamine was not measured to contradict the previous literature supporting 0.5 mg kg^−1^ ketamine for preemptive analgesia. Further studies should focus on postinsufflation response, clinical recovery score, and on long-term effects of ketamine in reducing chronic pain syndrome.

In conclusion, postinsufflation haemodynamic response and clinical recovery following laparoscopy were better in the ketamine group (0.25 mg kg^−1^) without any untoward side effects. Hence it can be considered as an optimal dose for preemptive analgesia in laparoscopic cholecystectomy and appendicectomy.

## Figures and Tables

**Table 1.** Demographic Data and Duration of Surgery of Study Population

**S. No**	**Variables**	**Groups**			*P*
**Group 1**	**Group 2**	**Group 3**
1	Age (Years)	33.2 ± 3.1	32.4 ± 2.4	30.6 ± 1.9	.748
2	Gender Male Female	12(48%) 13(52%)	10(40%) 15(60%)	11(44%) 14(56%)	.748
3	BMI (kg m−2)	26.5 ± 3.7	26.3 ± 3.7	26.2 ± 3.9	.884
4	Duration of surgery (minutes)	106.2 ± 3.0	100.2 ± 3.2	100.2 ± 3.2	.350

Values are presented as mean and standard deviation and gender differences in terms of proportion and percentages. BMI, Body Mass Index; *P* < .05 was considered to be significant. There was no significant difference in the demographic characteristics and duration of surgery in the study population.

**Table 2.** Distribution of Postoperative Side Effects Among Participants in the Study Groups

**S.No**	**Side effects**	**Group 1**	**Group 2**	**Group 3**	**Fisher’s exact ** *P* ** value**
1	Nausea 1 hour 2 hours >2 hours	3 (12%) 2 (8%) 0 (0%)	2 (8%) 0 (0%) 0 (0%)	1 (4%) 4 (16%) 0 (0%)	.351
2	Vomiting 1 hour 2 hours >2 hours	2 (8%) 1 (4%) 0 (0%)	2 (8%) 0 (0%) 0 (0%)	2 (8%) 0 (0%) 0 (0%)	1.000
3	Delirium 1 hour 2 hours >2 hours	0 (0%) 0 (0%) 0 (0%)	3 (12%) 0 (0%) 0 (0%)	8 (32%) 0 (0%) 0 (0%)	1.000

Values are represented in terms of percentages. Nausea and vomiting were more in Groups 1 and 3.

Delirium was more in Group 3.
